# Managing Hepatocellular Carcinoma in Children

**DOI:** 10.7759/cureus.26386

**Published:** 2022-06-28

**Authors:** Zunaira Shaukat, Mehwish Imtiaz, Rawaha Naqeeb, Iqtadar Seerat, Muhammad Atique, Faisal Dar

**Affiliations:** 1 Pediatric Oncology, Shaukat Khanum Memorial Cancer Hospital and Research Centre, Lahore, PAK; 2 Pediatric Gastroenterology, Pakistan Kidney and Liver Institute and Research Center, Lahore, PAK; 3 Pediatric Gastroenterology & Hepatology, Pakistan Kidney and Liver Institute and Research Center, Lahore, PAK; 4 Histopathology, Pakistan Kidney and Liver Institute and Research Center, Lahore, PAK; 5 Hepatobiliary Surgery, Pakistan Kidney and Liver Institute and Research Center, Lahore, PAK

**Keywords:** living donor liver transplant (ldlt), hepatocellular carcinoma, trans-arterial chemoembolization (tace), systemic chemotherapy, chemotherapy, children

## Abstract

Hepatocellular carcinoma (HCC) is a rare pediatric tumor. It differs from its adult counterpart in many ways like etiology, biological behavior, and association with cirrhosis. Treating HCC requires a multidisciplinary team involving pediatric gastroenterology, oncology, hepatobiliary surgery, and interventional radiology. This case series aims to describe presenting features and management plan of three children with HCC treated at a tertiary care liver transplant center in Pakistan.

## Introduction

Hepatocellular carcinoma (HCC) is the second most common liver tumor after hepatoblastoma (HB) in the pediatric population [1]. About two-thirds of cases of pediatric HCC occur between 15 and 19 years and account for 87% of liver tumors [2]. Pediatric HCC differs from its adult counterpart in etiological predisposition and biological behavior and is associated with a lower frequency of cirrhosis. Unlike adult HCC, which occurs often in the background of viral hepatitis, the majority of pediatric HCC occurs de novo [3]. In Asian children, a vast majority developed HCC in the context of chronic or congenital liver disease [4]. We aim to present a case series on the clinical presentation of pediatric HCC at our center and their outcomes.

## Case presentation

Case 1

A six-year-old boy, born to first cousins, presented with jaundice since the age of six weeks, associated with occasional vomiting, and dark-colored urine, not associated with clay-colored stools. The patient had severe itching from the age of six months and progressive abdominal distension from the age of two years. He had decompensation in the form of four episodes of epistaxis requiring packed cell transfusions, ascites, and jaundice. Family history was significant for the death of a sibling at 13 months of age with undiagnosed chronic liver disease. On examination, he had growth parameters at the fifth centile with deep jaundice, scratch marks on the body, hepatosplenomegaly, and signs of vitamin D deficiency. The labs of the patient are presented in Table 1.

**Table 1:** Baseline investigations of subject 1 at presentation

Investigation	Values
White blood cells	7.7 x 10.e 3/μl
Hemoglobin	8.7 g/dl
Platelets	227 x 10.e 6/μl
Total bilirubin	17.05 mg/dl
Direct bilirubin	11.7 mg/dl
Indirect bilirubin	5.34 mg/dl
Alanine transferase (ALT)	207 U/L
Aspartate transferase (AST)	673 U/L
Alkaline phosphatase	519 U/L
Albumin	3.19 g/dl
Gamma-glutamyl transferase (GGT)	32 U/L
Prothrombin time	12.9 seconds
International normalized ratio	1.2

**Figure 1:**
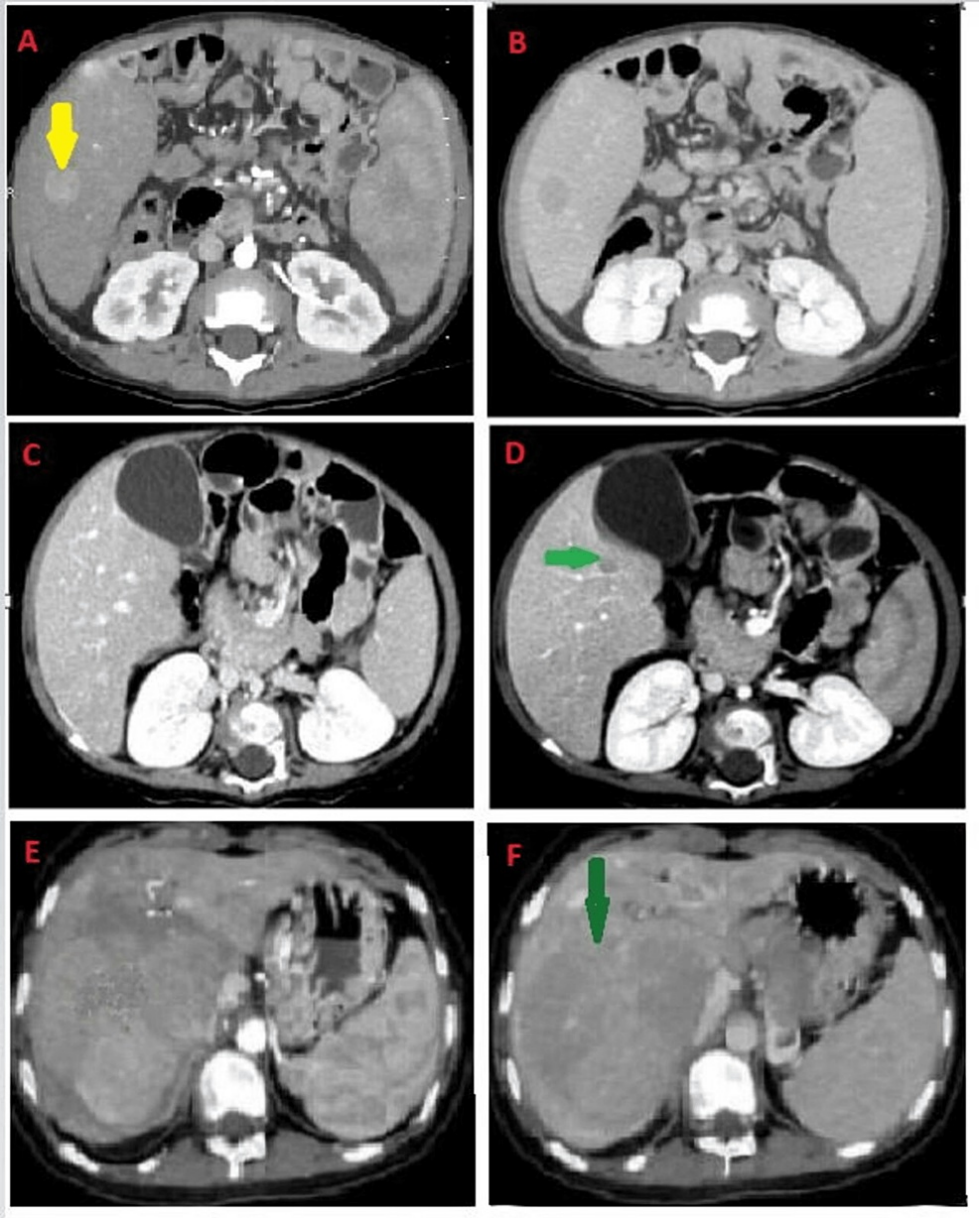
Triphasic CT of the abdomen of all three subjects (A) Triphasic CT of the abdomen of patient 1 showed an arterialized lesion in the right lobe of the liver, which showed washout on delayed images (B). (C and D) CT of the abdomen of patient 2 showed a 1 cm washed-out lesion in the caudate lobe (green arrow). (E) Triphasic CT of the abdomen of patient 3 showed hepatomegaly with multifocal intrahepatic well-defined enhancing liver masses with concomitant washout (F), favoring multifocal hepatocellular carcinomas. Hepatic veins were clearly patent.

A metabolic workup was done to rule out causes for neonatal hepatitis, which was inconclusive. An ultrasound of the abdomen was performed showing cirrhotic liver with F4 stage of hepatic fibrosis on elastogram. Triphasic CT (Figure 1) showed cirrhotic liver with undulating margins and an arterially enhancing focus in the segment VI, showing well-defined washout in the delayed phase suggestive of HCC (Figures 1A, 1B).

Genetic analysis was sent through a collaborative body to Centogene, Rostock, Germany, for a definitive diagnosis that confirmed progressive familial intrahepatic cholestasis (PFIC) type 2. A liver biopsy was performed that showed moderate inflammation composed of lymphocytes and plasma cells. Bile duct inflammation, sinusoidal dilatation, and ductular reaction were seen. Fibrosis, interface hepatitis, and focal necrosis were seen. Feathery degeneration and ballooning degeneration were present. Mallory-Denk bodies and multinucleated giant cell hepatocytes were seen. Alpha-fetoprotein (AFP) level was within the normal range.

The patient was discussed in a multidisciplinary meeting for an emergent liver transplant because of HCC development in the background of chronic liver disease. Currently, we are searching for a suitable donor. Meanwhile, the patient is being managed medically with regular follow-up with clinical examination, AFP, imaging, and liver function tests.

Case 2

**Figure 2:**
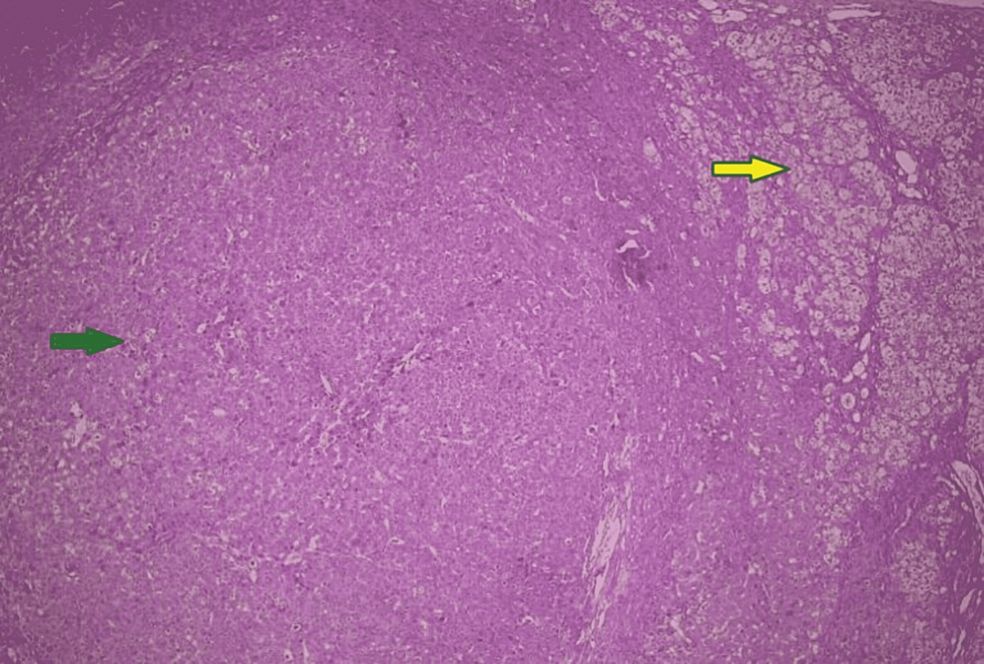
Photomicrograph of the resected liver of case 2 The green arrow shows nodules composed of sheets, pseudo acinar structures, and cords of cells with abundant cytoplasm and vesicular nuclei and prominent nucleoli suggestive of well-differentiated hepatocellular carcinoma. The background liver (yellow arrow) shows features of giant cell hepatitis with cholestasis in keeping with progressive familial intrahepatic cholestasis (PFIC) type 2.

Our second patient was a one-and-a-half-year-old boy, born of a consanguineous marriage, diagnosed with autosomal recessive PFIC type 2, based on a pathogenic variant in the ABCB11 gene. He presented with a history of jaundice since the age of four months, which was sudden and progressive associated with occasional fever and pigmented stools. It was associated with intense pruritus. Workup for obstructive and metabolic causes of neonatal jaundice was performed, which was inconclusive. His family history was significant for the death of two siblings with similar complaints at 11 and six months, respectively. So genetic analysis was sent through a collaborative body to Centogene, Rostock, Germany, which revealed a homozygous pathogenic variant in the ABCB11 gene. Both the parents were carriers for the heterozygous pathogenic variant in the ABCB11 gene. The patient was discussed in a multidisciplinary team (MDT) and planned for a live donor transplant. Baseline CT triphasic done as part of liver transplant workup showed an arterialized lesion in the caudate lobe that showed washout on delayed sequences (Figures 1C, 1D). AFP level was raised for his age. He underwent a live donor liver transplant and is doing well on immunosuppressive agents so far. Histopathology of his resected liver showed giant cell hepatitis and a focus of HCC in the caudate lobe (Figure 2). The pre- and post-transplant investigations of the patient are listed below in Table 2.

**Table 2:** Hematological parameters of subject 2 before and after liver transplant

Investigation	Pre-transplant	Post-transplant
White blood cells	12 x 10.e 3/μl	6.7 x 10.e 3/μl
Hemoglobin	11.6 g/dL	12.3 g/dL
Platelets	219 x 10.e 6/μl	417 x 10.e 6/μl
Total bilirubin	10.4 mg/dL	0.31 mg/dL
Direct bilirubin	7.07 mg/dL	0.12 mg/dL
Indirect bilirubin	3.34 mg/dL	0.19 mg/dL
Alanine transferase	104 U/L	23 U/L
Aspartate transferase	373 U/L	29 U/L
Gamma-glutamyl transferase	39 U/L	19 U/L
Albumin	3.7 g/dL	4.1 g/dL
Alkaline phosphatase	1235 U/L	427 U/L
Prothrombin time	30.6 seconds	12.2 seconds
International normalized ratio	2.3	0.94

Case 3

**Figure 3:**
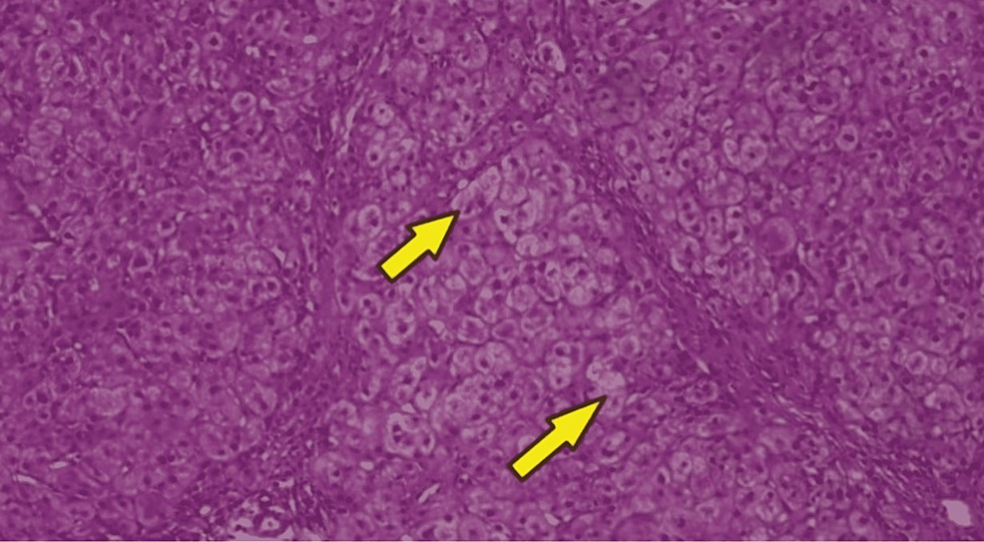
Biopsy Well-differentiated hepatocellular neoplasm with disturbed liver architecture and proliferation of hepatocytes. Few areas (yellow arrows) show hepatic plate thickness of >3 hepatocytes/plate. No portal tracts are seen. Areas that appeared as portal areas are devoid of bile ducts and have thick-walled blood vessels.

**Figure 4:**
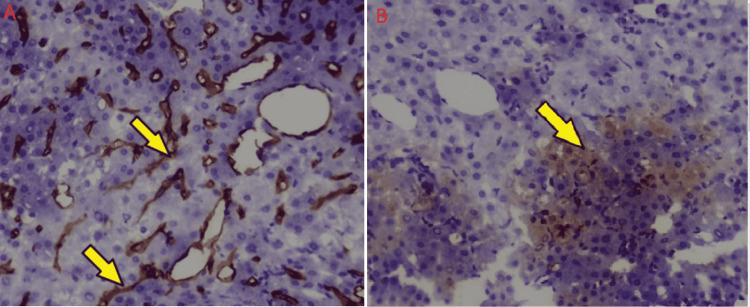
Immunohistochemistry (A) CD-34 complete endothelialization in portal areas. Portal areas are devoid of bile ducts showing only blood vessels. (B) Glypican 3 shows focal positivity suggestive of hepatocellular neoplasm in keeping with hepatocellular carcinoma.

Our third patient was a 14-year-old girl with no prior history of liver disease, developmentally normal, and presented incidentally with a liver mass detected on abdominal ultrasound. The patient had not achieved menarche for which an ultrasound of the abdomen was advised. She had vague symptoms of epigastric discomfort. Liver function tests were within normal range. CT triphasic of the abdomen was done that showed hepatomegaly with multifocal intrahepatic well-defined enhancing liver masses with concomitant washout, favoring multifocal HCCs. Hepatic veins were clearly patent (Figures 1E, 1F). Mass was biopsied and histopathology showed well-differentiated hepatocellular neoplasm with disturbed liver architecture and proliferation of hepatocytes. Few areas showed hepatic plate thickness of >3 hepatocytes/plate (Figure 3). No portal tracts were seen. Areas that appeared as portal areas were devoid of bile ducts and had thick-walled blood vessels (Figure 4A). Glypican 3 showed focal positivity suggesting HCC (Figure 4B). The patient was discussed in MDT and was planned for a liver transplant, as it was her only chance of survival. While searching for a suitable donor, transarterial chemoembolization (TACE) is done as a bridging therapy. The patient is doing well at the time of writing this manuscript.

## Discussion

Pediatric HCC is a biologically heterogeneous group of tumors. HCC may present in children with underlying liver pathology, as seen in our first two cases, but can also occur de novo like in our third patient. A timely liver transplant in these patients can improve survival.

Before the introduction of hepatitis B vaccination, hepatitis B virus was the major culprit for the development of HCC in children, documented in two-thirds of patients with pediatric HCC in one study [5]. Underlying liver diseases in which pediatric HCC has been reported include familial cholestatic syndromes (PFIC and Alagille syndrome), extrahepatic biliary atresia, and total parenteral nutrition, and is found in association with tyrosinemia, glycogenosis, neurofibromatosis, ataxia-telangiectasia, Fanconi anemia, and other constitutional and genetic abnormalities [6,7]. These associated causes are seen in about 25% of cases of pediatric HCC [7]. No data are available on causes and associations of pediatric HCC from Pakistan.

The mainstay of the pediatric HCC therapeutic strategy is the radical tumor resection, whether by hepatic resection or liver transplantation; nevertheless, the best surgical approaches, as well as the optimal neoadjuvant and adjuvant treatment, are still under debate [8]. Treatment modalities for HCC in children include surgery for localized tumors, chemo reduction followed by surgery for bigger tumors, and liver transplant for patients with a multifocal disease or very large tumors [9]. TACE may be used as a bridging therapy. A liver transplant gives the best survival for patients developing HCC in the background of chronic liver disease. Patients with HCC have previously been treated on the same protocols as patients with HB. In the INT0098 study, 46 HCC patients were enrolled. For the entire cohort, five-year event-free survival (EFS) was 19% (SD = 6%). Patients with stages I (n = 8), III (n = 25), and IV (n = 13) had five-year EFS of 88% (SD = 12%), 8% (SD = 5%), and 0%, respectively. Therefore, while children with resectable HCC had a good prognosis, those with advanced disease had a dismal outcome [10,11].

In patients with chronic liver disease due to cholestatic syndromes or constitutional and genetic abnormalities, close follow-up with periodic AFP, triphasic CT of the abdomen, and liver biopsies for suspicious lesions are indicated. These patients can develop HCC usually by the age of 10 years. In our case series, both patients with underlying liver disease developed HCC at very young ages; the first patient at six years and the other at the age of 13 months. With close surveillance, HCCs can be picked up early and treatment can have better outcomes. Patients with de novo HCC can be given a chance for surgical resection in limited disease, neoadjuvant chemotherapy, or TACE before going for a liver transplant. In case of multifocal disease or large tumors limited to the liver, a transplant can be offered, as was the case in our third patient.

The limitation of this case series is a very small number. As pediatric HCC is a rare entity, further multicenter studies across the country are needed to look upon epidemiology, management, and outcome.

## Conclusions

Management of pediatric HCC requires a multidisciplinary team. For children with resectable tumors, surgery remains the mainstay of treatment. Children with nonresectable tumors, which are localized, may benefit from liver transplantation. However, studies are required to evaluate and modify adult liver transplant criteria to be implemented in the pediatric population. Chemotherapy and TACE can be used as bridging therapy to definitive surgery. Research is needed for characterizing the molecular and genomic mechanisms of pediatric HCC to support the development of novel therapeutic agents and the implementation of personalized medicine.

## References

[1] Varol M, Kaçar E, Akın HK (2020). Accumulation of trace elements in muscle, gill and liver of fish species (Capoeta umbla and Luciobarbus mystaceus) in the Tigris River (Turkey), and health risk assessment. Environ Res.

[2] Khanna R, Verma SK (2018). Pediatric hepatocellular carcinoma. World J Gastroenterol.

[3] Czauderna P (2002). Adult type vs. childhood hepatocellular carcinoma—are they the same or different lesions? Biology, natural history, prognosis, and treatment. Med Pediatr Oncol.

[4] Ismail H, Broniszczak D, Kaliciński P (2009). Liver transplantation in children with hepatocellular carcinoma. Do Milan criteria apply to pediatric patients?. Pediatr Transplant.

[5] Moore SW, Millar AJ, Hadley GP (2004). Hepatocellular carcinoma and liver tumors in South African children: a case for increased prevalence. Cancer.

[6] Romano F, Stroppa P, Bravi M (2011). Favorable outcome of primary liver transplantation in children with cirrhosis and hepatocellular carcinoma. Pediatr Transplant.

[7] López-Terrada D, Alaggio R, de Dávila MT (2014). Towards an international pediatric liver tumor consensus classification: proceedings of the Los Angeles COG liver tumors symposium. Mod Pathol.

[8] Allan BJ, Wang B, Davis JS, Parikh PP, Perez EA, Neville HL, Sola JE (2014). A review of 218 pediatric cases of hepatocellular carcinoma. J Pediatr Surg.

[9] Czauderna P, Mackinlay G, Perilongo G (2002). Hepatocellular carcinoma in children: results of the first prospective study of the International Society of Pediatric Oncology group. J Clin Oncol.

[10] Trobaugh-Lotrario AD, Tomlinson GE, Finegold MJ, Gore L, Feusner JH (2009). Small cell undifferentiated variant of hepatoblastoma: adverse clinical and molecular features similar to rhabdoid tumors. Pediatr Blood Cancer.

[11] Katzenstein HM, Krailo MD, Malogolowkin MH (2002). Hepatocellular carcinoma in children and adolescents: results from the Pediatric Oncology Group and the Children's Cancer Group intergroup study. J Clin Oncol.

